# Supplementary material for: Drug Repositioning for HPV Clade-Specific Cervicouterine Cancer Using the OCTAD Pipeline
Source: Int J Mol Sci. 2025 Nov 20;26(22):11238. doi: 10.3390/ijms262211238 (PMC12653604; doi:10.3390/ijms262211238)
Supplement: Supplementary file 1 [file ijms-26-11238-s001.zip › Supplementary/Supplementary Material.pdf]

Supplementary Material

Table S1. Distribution of HPV genotypes in cervical cancer cohort samples.

HPV type	HPV clade A7	HPV clade A9
HPV16	0	166
HPV18	37	0
HPV31	0	7
HPV33	0	8
HPV35	0	6
HPV19	5	0
HPV45	20	0
HPV52	0	8
HPV58	0	7
HPV59	3	0
Total (n)	65	202

Table S2. Distribution of FIGO stages in cervical cancer between HPV clades.

FIGO stage	HPV clade A7	HPV clade A9	Total (n)
Stage I	35 (53.8%)	100 (49.5%)	135
Stage II	17 (26.2%)	43 (21.3%)	60
Stage III	10 (15.4%)	31 (15.3%)	41
Stage IV	2 (3.1%)	14 (6.9%)	16
Not Reported	1 (1.5%)	14 (6.9%)	15

Fisher's exact test: $p = 0.3794$.

Table S2 summarizes the distribution of FIGO stages [1] among cervical cancer samples stratified by HPV A7 and A9 clades. Values represent absolute counts followed by column-wise percentages within each clade. The comparison between HPV clades was assessed using Fisher's exact test (p = 0.3794), indicating no statistically significant difference in the distribution of clinical stages between clades.

Table S3. Distribution of histological types in cervical cancer between HPV clades.

Histological type	HPV clade A7	HPV clade A9	Total (n)
Adenocarcinoma (AC)	10 (15.4%)	26 (12.6%)	36
Adenosquamous carcinoma (ASC)	2 (3.1%)	1 (0.5%)	3
Squamous cell carcinoma (SCC)	53 (81.5%)	175 (86.9%)	228

Fisher's exact test: $p = 0.1665$

Table S3. shows the distribution of histological carcinoma types among cervical cancer samples classified by HPV A7 and A9 clades. Histological categories were grouped according to established histopathological classification criteria [2,3]. Values represent absolute counts followed by column wise percentages within each clade. The comparison between HPV clades was assessed using Fisher's exact test (p = 0.1665), indicating no statistically significant difference in the distribution of histological types between clades.

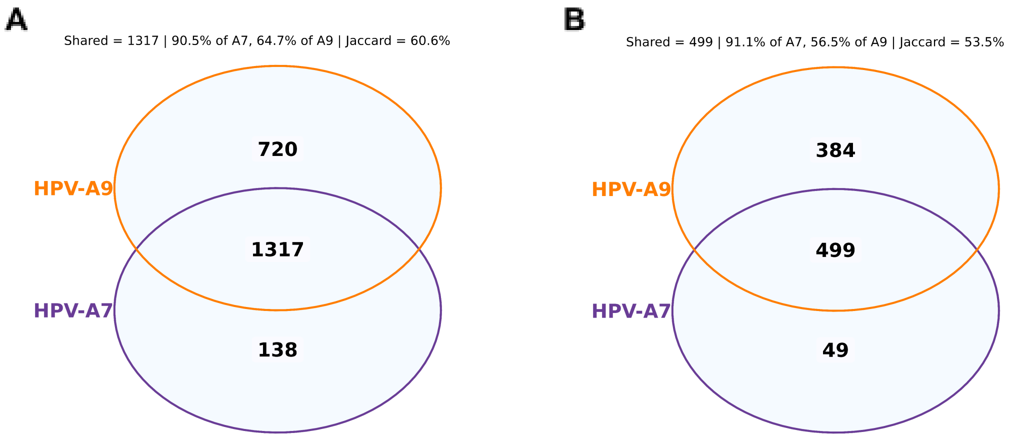

Supplementary Figure S1. Overlap of consensus differentially expressed genes (DEGs) between HPV clade A7 and HPV clade A9 cervical cancers.

Venn diagrams illustrating the overlap of consensus differentially expressed genes (DEGs) derived from the integration of three analytical methods (edgeR, DESeq2, and limma-voom) for HPV clade A7 and HPV clade A9 tumors, each compared with normal cervical controls. A) shows the overlap for underexpressed genes, and panel B) for overexpressed genes, each depicting the total number of DEGs identified per clade and their intersection. The values above the diagrams indicate the proportion of shared genes relative to each clade and the Jaccard index, which quantifies the similarity between both gene sets as the ratio of shared genes to the total number of unique genes across clades.

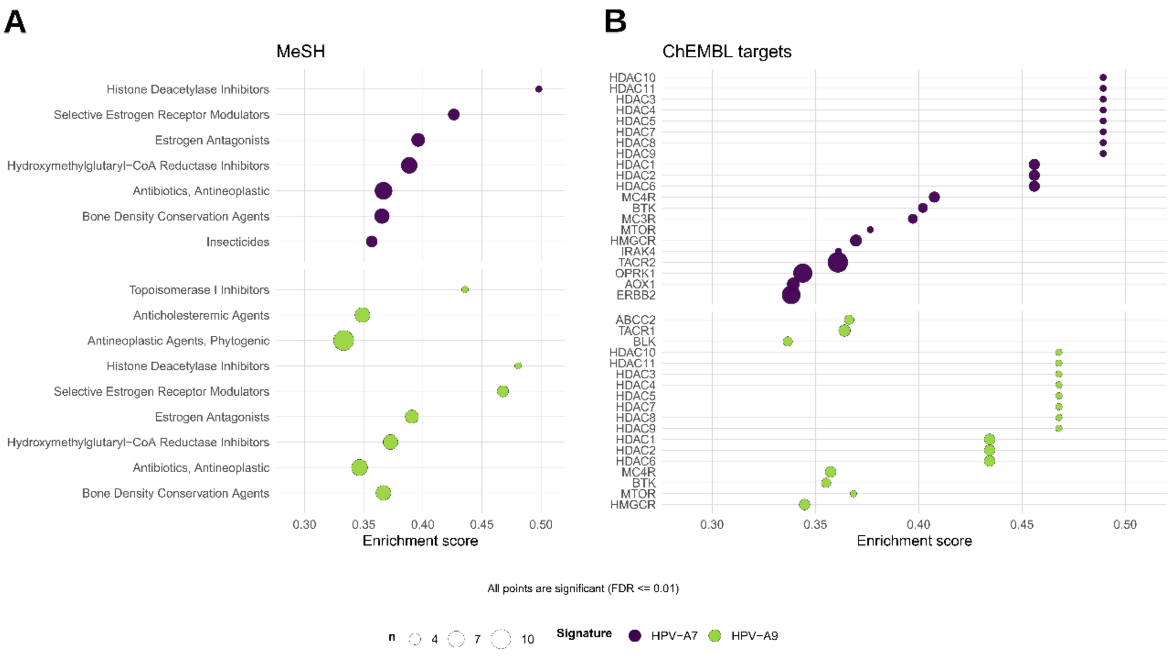

Figure S2. Drug set enrichment analysis of consensus sRGES results for FDA approved compounds in HPV clade A7 and HPV clade A9 cervical carcinomas.

A) Enrichment of pharmacological classes defined by MeSH (Medical Subject Headings).(B) Enrichment of molecular targets from ChEMBL For both graphs the x-axis indicates the enrichment score, while the size of the bubbles corresponds to the number of candidate drugs contributing to each category. Only categories with FDR ≤ 0.01 are shown. Green and purple denote enrichment for HPV clade A7 and HPV clade A9 signatures, respectively.

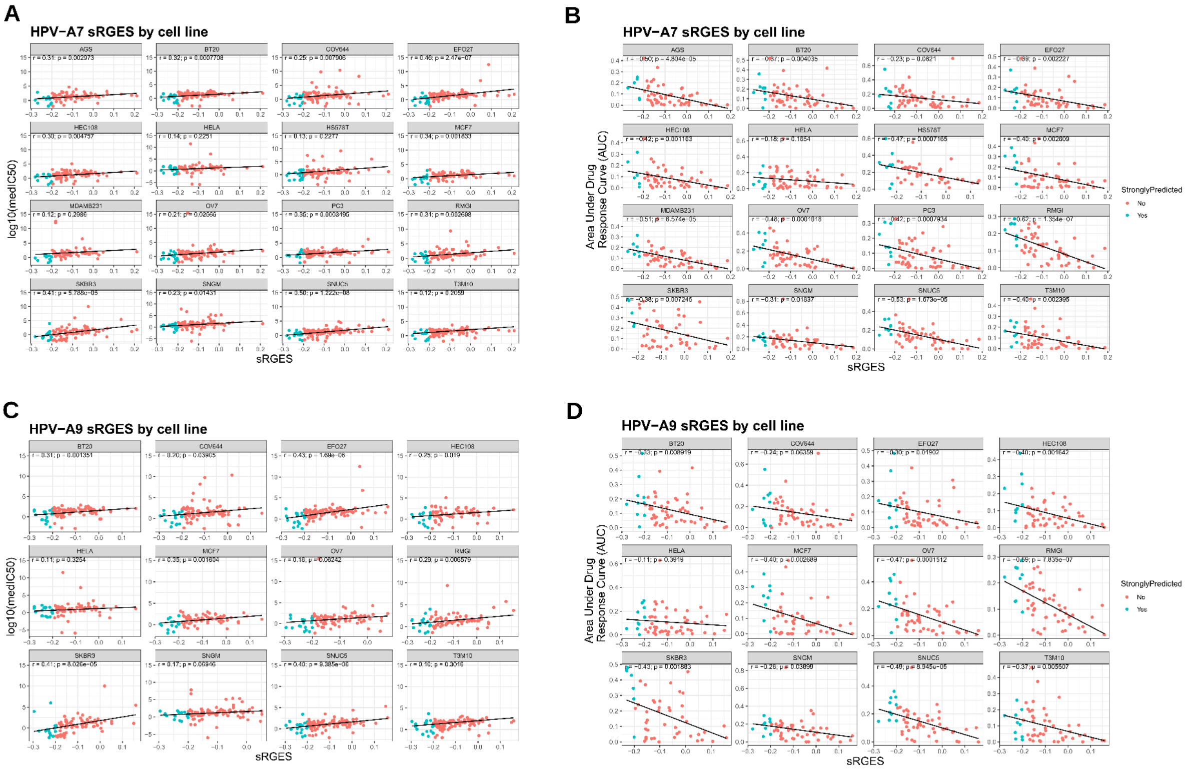

Figure S3. In-silico validation of consensus sRGES by cell line.
(A–B) HPV clade A7. (C–D) HPV clade A9. Scatter plots show, for each retained cell line (medcor > 0.30), the relationship between sRGES (x-axis) and AUC (recomputed) *(left panels A, C)* or log10(median IC50) *(right panels B, D)* for the set of drugs tested in common. The black line indicates the least-squares fit; each facet reports the correlation coefficient (*r*) and its P-value. Points highlighted as "Yes" in the legend correspond to compounds flagged as strongly predicted (RGES ≤ −0.20).

Table S4. Summary of experimental and clinical evidence supporting repositioned drug candidates in cervical cancer.

Drug name	MeSH class	Evidence type	Key finding
Pyrvinium		In vitro; in vivo	Wnt inhibition enhances chemotherapy response; antitumor activity in vivo with cisplatin [4].
Ivermectin	Anthelmintics	In vitro	Induces cell-cycle arrest and apoptosis in HeLa cells [5].
Closantel		In vivo	Anti-angiogenic; suppresses growth of cervical tumor xenografts in zebrafish [6].
Amodiaquine	Antimalarials	In vitro	Cytotoxicity in cervical cells. Suggests antitumor potential against cervical carcinoma [7].
Quinine		In vitro	Inhibits proliferation; induces apoptosis and suppresses AKT phosphorylation [8].
Methotrexate	Antimetabolite ; Folic Acid Antagonists	In vitro; In vivo	GOLM1 knockdown sensitizes cervical tumors to methotrexate (enhanced antitumor effect) [9].
Nifuroxazide	Antibiotic	in vitro	Decreased p-STAT3 and suppressed colony formation [10].

Penfluridol	Antipsychotic	In vitro	Increased the radiation sensitivity by inhibiting radiation-induced DNA break repair [11].
Palbociclib	Cyclin-Dependent Kinase Inhibitors	In vitro; in vivo	Antiproliferative effects on cervical cancer cells, especially when combined with the investigational drug SHetA2 [12].
Fulvestrant	Selective estrogen receptor modulators (SERMs)	In vivo	ER blockade reduces tumor growth in ER-positive cervical xenografts. [13]
Panobinostat	HDAC Inhibitors	In vitro	Strong antiproliferative/apoptotic effects; synergy with topoisomerase inhibitors [14]
Vorinostat		In vitro; In vivo; clinical phase II	HDAC inhibition suppresses cervical cancer growth; Phase II PEVOsq trial: combination of pembrolizumab + vorinostat in recurrent/metastatic cervical cancer showed an objective response rate (ORR) of 39% [15–17].
Simvastatin	HMG-CoA reductase inhibitor	In vitro; in vivo	Selective cytotoxicity enhances paclitaxel efficacy in vitro and in vivo [18] .
Pitavastatin		In vitro; in vivo	Strong apoptosis and G0/G1 arrest; tumor growth suppression in mice [19].
Bortezomib	Proteasome Inhibitors	In vitro	Induce radiosensitivity in cell lines [20,21].
Dasatinib	Protein Kinase Inhibitors	In vitro	Enhances paclitaxel/oxaliplatin activity; increases apoptosis via Src pathway inhibition in cervical adenocarcinoma cells [22].
Raloxifene	Selective estrogen receptor modulator (SERM)	In vitro	In HPV16^{+} CaSki cervical cancer cells, raloxifene (an estrogen antagonist) blocked 17β-estradiol's effects – inhibiting estrogen-driven cell growth and HPV E6/E7 oncogene expression [23].
Duloxetine	Serotonin-norepinephrine reuptake inhibitors (SNRIs)	In vitro	Induces apoptosis/autophagy and inhibits Akt [24].
SN-38	Topoisomerase I Inhibitors	Clinical phase II	Sacituzumab govitecan showed antitumor activity with manageable toxicity (NCT05119907) ; irinotecan+S-1 was active in platinum-pretreated disease (ORR 29%, PFS 3 mo, OS 9 mo) [25,26] .
Etoposide	Topoisomerase II Inhibitors	In vitro; Clinical case report	Triggers apoptosis in HeLa; backbone with cisplatin for neuroendocrine cervical carcinoma [27,28].

References

1. Matsuo, K.; Machida, H.; Mandelbaum, R.S.; Konishi, I.; Mikami, M. Validation of the 2018 FIGO Cervical Cancer Staging System. *Gynecol. Oncol.* **2019**, *152*, 87–93, doi:10.1016/j.ygyno.2018.10.026.
2. Höhn, A.K.; Brambs, C.E.; Hiller, G.G.R.; May, D.; Schmoeckel, E.; Horn, L.-C. 2020 WHO Classification of Female Genital Tumors. *Geburtshilfe Frauenheilkd.* **2021**, *81*, 1145–1153, doi:10.1055/a-1545-4279.
3. Meijer, C.J.L.M.; Steenbergen, R.D.M. Gynaecological Cancer: Novel Molecular Subtypes of Cervical Cancer - Potential Clinical Consequences. *Nat. Rev. Clin. Oncol.* **2017**, *14*, 397–398, doi:10.1038/nrclinonc.2017.52.

4. Xu, H.; Wang, Z.; Xu, L.; Mo, G.; Duan, G.; Wang, Y.; Sun, Z.; Chen, H. Targeting the eIF4E/β-Catenin Axis Sensitizes Cervical Carcinoma Squamous Cells to Chemotherapy. *Am. J. Transl. Res.* **2017**, *9*, 1203–1212.
5. Zhang, P.; Zhang, Y.; Liu, K.; Liu, B.; Xu, W.; Gao, J.; Ding, L.; Tao, L. Ivermectin Induces Cell Cycle Arrest and Apoptosis of HeLa Cells via Mitochondrial Pathway. *Cell Prolif.* **2019**, *52*, e12543, doi:10.1111/cpr.12543.
6. Zhu, X.-Y.; Xia, B.; Liu, H.-C.; Xu, Y.-Q.; Huang, C.-J.; Gao, J.-M.; Dong, Q.-X.; Li, C.-Q. Closantel Suppresses Angiogenesis and Cancer Growth in Zebrafish Models. *Assay Drug Dev. Technol.* **2016**, *14*, 282–290, doi:10.1089/adt.2015.679.
7. Islam, S.; Shahzad, S.A.; Hassan Bin Asad, M.H.; Mannan, A. Novel Amodiaquine Analogues to Treat Cervical Cancer and Microbial Infection in the Future. *Future Med. Chem.* **2023**, *15*, 2165–2179, doi:10.4155/fmc-2023-0245.
8. El-Mesery, M.; Seher, A.; El-Shafey, M.; El-Dosoky, M.; Badria, F.A. Repurposing of Quinoline Alkaloids Identifies Their Ability to Enhance Doxorubicin-Induced Sub-G0/G1 Phase Cell Cycle Arrest and Apoptosis in Cervical and Hepatocellular Carcinoma Cells. *Biotechnol. Appl. Biochem.* **2021**, *68*, 832–840, doi:10.1002/bab.1999.
9. Li, R.M.; Nai, M.M.; Duan, S.J.; Li, S.X.; Yin, B.N.; An, F.; Zhai, Y.Q.; Liu, J.; Chu, Y.R.; Yu, Y.; et al. Down-Expression of GOLM1 Enhances the Chemo-Sensitivity of Cervical Cancer to Methotrexate through Modulation of the MMP13/EMT Axis. *Am. J. Cancer Res.* **2018**, *8*, 964–980.
10. Zhang, J.; Gao, Y. Long Non-Coding RNA MEG3 Inhibits Cervical Cancer Cell Growth by Promoting Degradation of P-STAT3 Protein via Ubiquitination. *Cancer Cell Int.* **2019**, *19*, 175, doi:10.1186/s12935-019-0893-z.
11. Du, J.; Shang, J.; Chen, F.; Zhang, Y.; Yin, N.; Xie, T.; Zhang, H.; Yu, J.; Liu, F. A CRISPR/Cas9-Based Screening for Non-Homologous End Joining Inhibitors Reveals Ouabain and Penfluridol as Radiosensitizers. *Mol. Cancer Ther.* **2018**, *17*, 419–431, doi:10.1158/1535-7163.MCT-17-0090.
12. Kennedy, A.L.; Rai, R.; Isingizwe, Z.R.; Zhao, Y.D.; Lightfoot, S.A.; Benbrook, D.M. Complementary Targeting of Rb Phosphorylation and Growth in Cervical Cancer Cell Cultures and a Xenograft Mouse Model by SHetA2 and Palbociclib. *Cancers* **2020**, *12*, 1269, doi:10.3390/cancers12051269.
13. Ramachandran, B.; Murhekar, K.; Sundersingh, S. SERMs Suppresses the Growth of ERα Positive Cervical Cancer Xenografts through Predominant Inhibition of Extra-Nuclear ERα Expression. *Am. J. Cancer Res.* **2021**, *11*, 3335–3353.
14. Sheng, B.; Wang, W.; Xia, D.; Qu, X. Panobinostat (LBH589) Combined with AM1241 Induces Cervical Cancer Cell Apoptosis through Autophagy Pathway. *BMC Pharmacol. Toxicol.* **2023**, *24*, 45, doi:10.1186/s40360-023-00686-7.
15. Borcoman, E.; Cabarrou, B.; Francisco, M.; Bigot, F.; Ghiringhelli, F.; Vansteene, D.; Legrand, F.; Halladjian, M.; Dupain, C.; Le Saux, O.; et al. Efficacy of Pembrolizumab and Vorinostat Combination in Patients with Recurrent and/or Metastatic Squamous Cell Carcinomas: A Phase 2 Basket Trial. *Nat. Cancer* **2025**, *6*, 1370–1383, doi:10.1038/s43018-025-01004-2.
16. Pan, B.; Yin, S.; Peng, F.; Liu, C.; Liang, H.; Su, J.; Hsiao, W.L.W.; Cai, Y.; Luo, D.; Xia, C. Vorinostat Targets UBE2C to Reverse Epithelial-Mesenchymal Transition and Control Cervical Cancer Growth through the Ubiquitination Pathway. *Eur. J. Pharmacol.* **2021**, *908*, 174399, doi:10.1016/j.ejphar.2021.174399.
17. Huang, Z.; Peng, S.; Knoff, J.; Lee, S.Y.; Yang, B.; Wu, T.-C.; Hung, C.-F. Combination of Proteasome and HDAC Inhibitor Enhances HPV16 E7-Specific CD8+ T Cell Immune Response and Antitumor Effects in a Preclinical Cervical Cancer Model. *J. Biomed. Sci.* **2015**, *22*, 7, doi:10.1186/s12929-014-0111-1.

18. Pan, Q.; Xu, J.; Ma, L. Simvastatin Enhances Chemotherapy in Cervical Cancer via Inhibition of Multiple Prenylation-Dependent GTPases-Regulated Pathways. *Fundam. Clin. Pharmacol.* **2020**, *34*, 32–40, doi:10.1111/fcp.12479.

19. Hacıseyitoğlu, A.Ö.; Doğan, T.Ç.; Dilsiz, S.A.; Canpınar, H.; Eken, A.; Bucurgat, Ü.Ü. Pitavastatin Induces Caspase-Mediated Apoptotic Death through Oxidative Stress and DNA Damage in Combined with Cisplatin in Human Cervical Cancer Cell Line. *J. Appl. Toxicol. JAT* **2024**, *44*, 623–640, doi:10.1002/jat.4565.

20. Cui, H.; Qin, Q.; Yang, M.; Zhang, H.; Liu, Z.; Yang, Y.; Chen, X.; Zhu, H.; Wang, D.; Meng, C.; et al. Bortezomib Enhances the Radiosensitivity of Hypoxic Cervical Cancer Cells by Inhibiting HIF-1α Expression. *Int. J. Clin. Exp. Pathol.* **2015**, *8*, 9032–9041.

21. Sun, C.; Meng, X.; Cui, X.; Liang, S.; Sun, J.; Zhang, B.; Cui, Y.; Zhao, Y.; Chen, N.; Tian, K.; et al. APG-115 Synergizes with Bortezomib to Induce Apoptosis in Cervical Cancer Cells. *Anticancer. Drugs* **2025**, *36*, 637–647, doi:10.1097/CAD.0000000000001735.

22. Takiguchi, E.; Nishimura, M.; Mineda, A.; Kawakita, T.; Abe, A.; Irahara, M. Growth Inhibitory Effect of the Src Inhibitor Dasatinib in Combination with Anticancer Agents on Uterine Cervical Adenocarcinoma Cells. *Exp. Ther. Med.* **2017**, *14*, 4293–4299, doi:10.3892/etm.2017.5061.

23. Ma, J.-Q.; Wang, X.-H.; Tang, L.-P.; Chen, X.-W.; Lou, G. Raloxifene Suppress Proliferation-Promoting Function of Estrogen in CaSKi Cervical Cells. *Int. J. Clin. Exp. Med.* **2015**, *8*, 5571–5575.

24. Nikolic, I.; Lazovic, A.; Stanisavljevic, I.; Andjelkovic, M.; Popovic, S.; Pavlovic, S.; Jurisevic, M.; Mitrovic, M. Duloxetine's Potential Dual Antitumor and Immunomodulatory Role in Apoptosis and Autophagy Signaling Pathways in Cancer: In Vitro and In Vivo Evidence. *Eur. J. Pharm. Sci. Off. J. Eur. Fed. Pharm. Sci.* **2025**, *212*, 107165, doi:10.1016/j.ejps.2025.107165.

25. Mabuchi, S.; Yokoi, E.; Shimura, K.; Komura, N.; Matsumoto, Y.; Sawada, K.; Isobe, A.; Tsutsui, T.; Kitada, F.; Kimura, T. A Phase II Study of Irinotecan Combined with S-1 in Patients with Advanced or Recurrent Cervical Cancer Previously Treated with Platinum Based Chemotherapy. *Int. J. Gynecol. Cancer* **2019**, *29*, 474–479, doi:10.1136/ijgc-2018-000070.

26. An, J.; Li, G.; Zhang, Y.; Feng, M.; Kong, W.; Jiang, H.; Luo, S.; Li, W.; Xu, C.; Han, L.; et al. Sacituzumab Govitecan in Chinese Patients with Recurrent/Metastatic Cervical Cancer: Results from the Phase 2 EVER-132-003 Basket Study (NCT05119907). *Gynecol. Oncol.* **2025**, *202*, 33–40, doi:10.1016/j.ygyno.2025.09.001.

27. Jin, H.; Suh, D.-S.; Kim, T.-H.; Yeom, J.-H.; Lee, K.; Bae, J. IER3 Is a Crucial Mediator of TAp73β-Induced Apoptosis in Cervical Cancer and Confers Etoposide Sensitivity. *Sci. Rep.* **2015**, *5*, 8367, doi:10.1038/srep08367.

28. Pei, X.; Xiang, L.; Ye, S.; He, T.; Cheng, Y.; Yang, W.; Wu, X.; Yang, H. Cycles of Cisplatin and Etoposide Affect Treatment Outcomes in Patients with FIGO Stage I-II Small Cell Neuroendocrine Carcinoma of the Cervix. *Gynecol. Oncol.* **2017**, *147*, 589–596, doi:10.1016/j.ygyno.2017.09.022.
